# Pinpoint, annular macules on scalp of a patient with systemic lupus erythematosus and alopecia

**DOI:** 10.1016/j.jdcr.2026.04.018

**Published:** 2026-04-20

**Authors:** Rishi M. Ray, Aref Moshayedi, Kimberly S. Salkey

**Affiliations:** a School of Medicine, Virginia Commonwealth University, Richmond, Virginia; b Department of Dermatology, Virginia Commonwealth University, Richmond, Virginia

**Keywords:** dermoscopy, trichoscopy, hair, alopecia, postinflammatory hyperpigmentation

## Case Description

**Fig 1:**
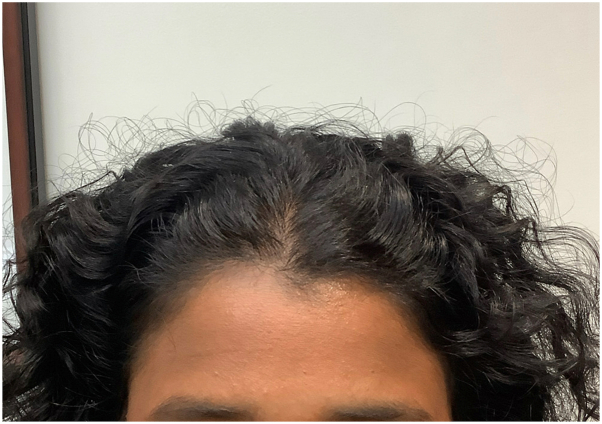
Clinical image. Decreased hair density in the frontal scalp.

**Fig 2:**
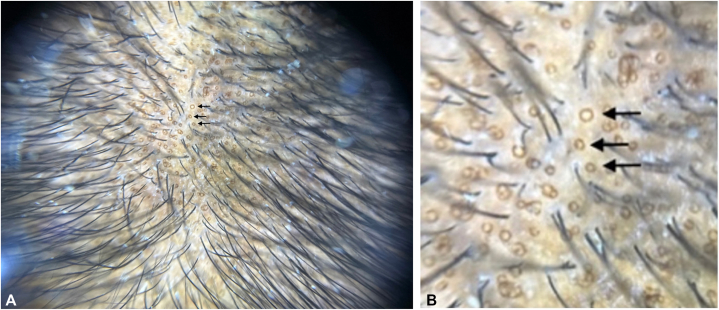
Dermatoscopic image trichoscopic view showing hyperpigmented, pinpoint, annular macules (*black arrows*).

A 31-year-old female with a history of well-controlled systemic lupus erythematosus (SLE) on oral hydroxychloroquine was referred to the dermatology department for progressive, diffuse hair loss primarily affecting the frontal scalp. The patient reported hair shedding for over a decade, which recently increased in severity over the last 1.5 y. No current symptoms of SLE or recent stressors were reported. Physical examination revealed diffusely decreased hair density in the fronto-temporal and crown regions with no discrete patches of hair loss (Fig 1). Notably, trichoscopy revealed multiple prominent hyperpigmented, pinpoint, annular macules on the affected areas of alopecia, without erythema, perifollicular scale, tubular casts, or structureless white areas. (Fig 2).

**Question: Which of the following is the most likely cause for the annular macules appreciated on trichoscopy?**

**A.** Sebum-filled hair follicles devoid of hair shafts associated with androgenic alopecia
**B.** Lichen planus pigmentosus presenting concomitantly with SLE
**C.** Postinflammatory hyperpigmentation associated with microneedling roller device
**D.** Lichen planopilaris presenting concomitantly with SLE
**E.** Postinflammatory hyperpigmentation associated with prior SLE activity

**Answer:**

**C.** Postinflammatory hyperpigmentation associated with microneedling roller device.

## Discussion

Upon prompting, the patient reported using a microneedling roller device 1 week prior to stimulate scalp blood flow and hair growth. The patient was diagnosed with female pattern hair loss likely unrelated to her SLE.

The lesions were limited to areas of microneedling, following a uniform distribution consistent with the microneedling device pattern, with a targetoid, nonfollicularly based pigment pattern consistent with postinflammatory hyperpigmentation (PIH). The absence of erythema, perifollicular hemorrhages, black dots, scaling, tubular casts, or structureless white areas reduced the likelihood of trichotillomania-associated PIH or intrinsic scalp disease.

The presence of the discrete, brown, pinpoint, annular lesions were consistent with PIH caused by microneedling and interestingly were a notable exogenous confounder in this patient’s diagnosis. Androgenic alopecia and alopecia areata are both nonscarring alopecias that present on trichoscopy with characteristic yellow dots, which are sebum-filled hair follicles devoid of hair shafts.^1^^,^^2^ These diagnostic trichoscopic signs may be mimicked or masked by the presentation of PIH lesions caused by microneedling and potentially result in misdiagnosis or delayed diagnosis of androgenic alopecia or alopecia areata.

Microneedling, or collagen induction therapy, is an established method for creating cutaneous micro-injuries to stimulate collagen and elastin production. Microneedling devices, or dermarollers, are now commonly sold for use at home and marketed to treat wrinkles and hair loss.^3^ Given the public access to at-home microneedling and their marketing for hair loss treatment, it is important to recognize associated PIH in patients presenting with hair loss when diagnosing alopecia. Careful history must be elicited as patients often do not initially report using such devices. Transient postinflammatory hyperpigmentation is the most commonly reported side effect of microneedling, and PIH lesions usually resolve within 2 wk, with darker skin tones experiencing a longer time to PIH resolution.^4^ To minimize misdiagnosis and delayed diagnosis in patients with various types of alopecia, dermatologists should be aware of this unique exogenous confounder. As always, clinical correlation is paramount.

### Declaration of generative AI and AI-assisted technologies in the writing process

No AI tools were used in the preparation of this manuscript.

## Conflicts of interest

None disclosed.
